# Impaired striatal glutamate/GABA regulation in violent offenders with antisocial personality disorder and psychopathy

**DOI:** 10.1038/s41380-024-02437-4

**Published:** 2024-02-07

**Authors:** John Tully, Andreia C. Pereira, Arjun Sethi, Julia Griem, Ben Cross, Steve CR Williams, Robert James Blair, Declan Murphy, Nigel Blackwood

**Affiliations:** 1 Academic Unit of Mental Health and Clinical Neurosciences, School of Medicine, University of Nottingham, Jubilee Campus, University of Nottingham, Wollaton Rd, Lenton, Nottingham, NG8 1BB United Kingdom; 2 Department of Forensic and Neurodevelopmental Sciences, Institute of Psychiatry, Psychology and Neuroscience, Kings College London, 16 De Crespigny Park, London, SE5 8AF United Kingdom; 3 Centre for Neuroimaging Sciences, Institute of Psychiatry, Psychology and Neuroscience, Kings College London, 16 De Crespigny Park, London, SE58AF United Kingdom; 4 Child and Adolescent Mental Health Centre, Mental Health Services, Capital Region of Denmark, Copenhagen, Denmark

**Keywords:** Neuroscience, Molecular biology

## Abstract

Men with antisocial personality disorder (ASPD) with or without psychopathy (+/−P) are responsible for most violent crime in society. Development of effective treatments is hindered by poor understanding of the neurochemical underpinnings of the condition. Men with ASPD with and without psychopathy demonstrate impulsive decision-making, associated with striatal abnormalities in functional neuroimaging studies. However, to date, no study has directly examined the potential neurochemical underpinnings of such abnormalities. We therefore investigated striatal glutamate: GABA ratio using Magnetic Resonance Spectroscopy in 30 violent offenders (16 ASPD-P, 14 ASPD + P) and 21 healthy non-offenders. Men with ASPD +/− P had a significant reduction in striatal glutamate : GABA ratio compared to non-offenders. We report, for the first time, striatal Glutamate/GABA dysregulation in ASPD +/− P, and discuss how this may be related to core behavioral abnormalities in the disorders.

## Introduction

A small group of men meet diagnostic criteria for Conduct Disorder (CD) in childhood and Antisocial Personality Disorder (ASPD) in adulthood. They are responsible for most violent crime [1, 2], resulting in a substantial negative impact on society [3, 4]. One-third of this group exhibit callous-unemotional traits in childhood [5, 6] and meet additional diagnostic criteria for psychopathy (ASPD + P) in adulthood [7]. The ASPD + P group have an earlier onset and greater density of offending behaviors [8] and respond less well to therapeutic strategies [9, 10] than those without psychopathy (ASPD-P).

Such life course persistent antisocial behavior is associated with dysfunctional empathic processing and impaired decision making. While empathic processing deficits, such as a deficient response to others’ fear [11–13] and distress [14], appear to be relatively specific to ASPD + P, it is less clear to what extent decision-making abnormalities are shared by or specific to ASPD + P and/or ASPD-P. Most studies in antisocial adults have focused on those with ASPD + P, who demonstrate abnormalities in tasks measuring passive avoidance [15], extinction [16], and reversal learning [17–19]. However, at least one set of neuropsychological studies has suggested that violent offenders with ASPD + P and ASPD-P demonstrate similar deficits in reversal learning, decision-making under risk, and stimulus-reinforcement-based decision-making [20]. Both groups of violent offenders failed to learn from punishment cues, to change their behavior in the face of changing contingencies, and made poorer quality decisions despite longer periods of deliberation.

The striatum, the principal input structure of the basal ganglia, may be a key neural substrate of such decision-making deficits. Both the ventral striatum (nucleus accumbens and olfactory tubercle) and the dorsal striatum (caudate and putamen) play crucial, partly dissociable roles in decision-making in healthy populations. The ventral striatum is thought to primarily process social reward and to underpin the reinforcement learning which helps to predict future outcomes [21]. The dorsal striatum predominantly mediates choice impulsivity, evaluating action-contingent outcomes to better select future goal-directed actions [22]. The function of the striatum is controlled by a complex array of neurotransmitters and neuromodulators [23, 24]. Dopamine, released in striatum by long-range axons arising from midbrain ventral tegmental area (VTA) and substantia nigra pars compacta (SNc), is thought to drive reinforcement learning by encoding reward prediction error (RPE), the difference between experienced and expected reward, doing so by regulating multiple aspects of neuronal and synaptic function [25–28].

The mesostriatal dopaminergic reward prediction error signal operates in the context of, and interacts with, striatal excitatory/inhibitory (E/I) balance. The critical balance between the strength of excitatory and inhibitory transmission is responsible for setting the local level of striatal excitability and is determined by glutamate and GABA. Hence, regulation of dopaminergic function by glutamate-GABA mediated E/I balance is likely a critical factor in controlling the ability of striatal circuits to optimize their computational reward behavior functions. Evidence from preclinical studies provides insights into how this occurs at a molecular level. Striatal cholinergic interneurons reduce or ‘pause’ their firing in response to both rewarding and punishing stimuli over the course of learning, serving to modulate reinforcement learning [29]. This cholinergic signal is in turn regulated by thalamo- and corticostriatal glutamatergic inputs [30]. Striatal dopamine release is inhibited by GABA-A or GABA-B receptor agonists, enhanced by of GABA-A and GABA-B receptor antagonists acting together, and enhanced by GABA-B antagonists acting alone [31].

Impaired valence-based modulation of dopaminergic prediction error signaling in the striatum has been hypothesized to underpin the abnormalities of reinforcement-based decision making observed in ASPD +/− P [32]. Functional MRI studies have provided evidence for ventral and dorsal striatal abnormalities in antisocial groups across the lifespan. In childhood, youths with Disruptive Behavior Disorders (DBD: Conduct Disorder and Oppositional Defiant Disorder, both precursors of ASPD in adulthood) show decreased ventral striatal response during reward anticipation [33], reduced responsiveness to positive prediction errors and increased responsiveness to negative prediction errors within the dorsal striatum during feedback [34], and reduced dorsal striatal response to early stimulus-reinforcement exposure [35]. Youths with persistent DBD demonstrate reduced responsivity in the ventral striatum during reward outcome processing, compared to youths who had desisted from DBD and healthy youth [36]. In adulthood, high psychopathy scores in incarcerated men are related to stronger subjective value-related activity within the ventral striatum during inter-temporal choice [37] but a reduced response in the ventral striatum to monetary loss [38]. Other studies in male prisoners with psychopathy (or high levels of psychopathic traits) have found increased responses to reward anticipation in the ventral striatum [37, 39]. Taken together, the evidence suggests that striatal dysfunction in both reward anticipation and reinforcement may underpin dysfunctional decision-making in antisocial populations. To date, however, there is insufficient evidence in youth and adult samples to determine whether neural correlates of decision-making deficits are shared across all antisocial individuals or are unique to severe and persistent forms of DBD in youth and psychopathy in adulthood.

These functional neuroanatomical studies were important first steps, but are ‘mute’ with respect to the potential neurochemical underpinnings of the decision making abnormalities. To date, no study to our knowledge has examined the role of glutamate/GABA mediated E/I balance in ASPD +/− P. Furthermore, despite the importance of striatal deficits in decision-making processes in antisocial populations, no study has specifically explored E/I balance in the striatum in antisocial groups. Hence, in a group of violent offenders with ASPD +/− P, and healthy non-offenders, we measured the striatal glutamate : GABA ratio using proton magnetic resonance spectroscopy (^1^H-MRS). We hypothesized that the glutamate : GABA ratio would be dysregulated in both ASPD + P and ASPD − P compared to healthy non-offenders, given the similar decision making abnormalities previously demonstrated in the violent offending groups.

## Methods

### Participants and assessment

Between September 2017 and March 2020, we enrolled 51 men (21 healthy non-offenders, 30 offenders with antisocial personality disorder with (*n* = 14) or without (*n* = 16) psychopathy), aged 20–58 years, with an IQ in the normal range as defined by the Wechsler Abbreviated Scale of Intelligence (WASI-II). [40] Offenders with convictions for violent crimes (murder, rape, attempted murder, grievous and actual bodily harm) who met DSM-5 criteria for antisocial personality disorder (Structured Clinical Interview for DSM-5 Personality Disorders (SCID-5 PD; [41]) were recruited via the National Probation Service of England and Wales and local forensic personality disorder services. Healthy non-offenders were recruited from the general population using online adverts and fliers in job centers and local recreational centers. All participants completed diagnostic (Strucured Clinical Interview for DSM-5 Disorders (SCID-5-RV)) [42] and Psychopathy Checklist- Revised (PCL-R; [43]) interviews and authorized access to their criminal records. A cross-cultural validation study [44] of the PCL-R demonstrated that cut off scores for psychopathy in men vary between North America (30 out of a possible 40 points) and Europe (25 out of a possible 40 points). In line with previous research in UK samples [45, 46], we used a score of 25 as the threshold for psychopathy in this English population. We calculated total, factor 1 and factor 2 PCL-R scores for all participants. Factor 1 scores are a total of facet 1 (interpersonal traits, such as pathological lying) plus facet 2 traits (affective traits, such as lack of empathy), while factor 2 scores are a total of facet 3 (antisocial lifestyle traits, such as impulsivity) plus facet 4 traits (overt antisocial behaviors, such as criminal versatility). Exclusion criteria were: history of major mental disorders (bipolar 1, bipolar 2, major depression or psychotic disorders) or self-reported neurological disorders, head injury resulting in loss of consciousness for 1 h or longer, severe visual or hearing impairments, or contraindications to MRI.

After receiving a complete description of the study, all participants provided written consent. Ethical approval was obtained from the national UK research authority (National Health Service Health Research Authority Research and Ethics Committee, project number 15/LO/1083). All assessments were conducted by an experienced forensic psychiatrist (JT). Participants completed the reactive-proactive aggression questionnaire [47]. On the day of each MRI scan, participants provided a urine sample to assess for substance misuse.

### Statistical analysis of demographic and psychometric data

Continuous variables were checked for normal distribution using the Shapiro–Wilk test (all datasets smaller than 2000 elements) and analyzed using independent sample t tests (for healthy non-offenders vs All ASPD, post-hoc comparisons of ASPD-P and ASPD + P). Categorical variables were analyzed using Chi-Squared tests, or Fisher’s exact test where there were less than 5 subjects in a cell. Significance level of *p* < 0.05 was used in all instances, with Bonferroni correction for multiple comparisons where indicated. All analyses were conducted using Statistical Package for Social Sciences, Version 25.0 for Windows [48]. Graphs displaying results were produced using GraphPad Prism version 7 for Mac (GraphPad Software, La Jolla, CA, USA, www.graphpad.com).

### Proton magnetic resonance spectroscopy (^1^H-MRS)

#### Scanning session and data acquisition

Participants underwent a ^1^H-MRS scan in a 3 Tesla General Electric MR750 Discovery scanner using a 32-channel head coil. A T_1_-weighted high resolution sagittal ADNI Go Spoiled Gradient Recalled (SPGR) anatomical with repetition time (TR) = 7.312 ms, echo time (TE) = 3.016 ms, inversion time (TI) = 400 ms, flip angle (FA) 11°, field of view 270 mm, 256 ×256 matrix, 196 slices, voxel dimensions: 1.055 ×1.055 ×1.2 mm was used for spectroscopy voxel positioning and further voxel tissue segmentation. A single-voxel (35 ×30 ×25 mm) was positioned to include the left striatum region of interest (ROI) using the anatomical scan (see Supplementary Fig. 1) and a Mescher-Garwood Point-Resolved Spectroscopy (MEGA-PRESS) [49] sequence (TR = 2000 ms, TE = 68 ms, bandwidth = 5 kHz; number of data points = 4096; 320 averages (160 ON and 160 OFF); phase cycle length of two; FA 90° (excitation pulses); CHESS water suppression) was used to quantify GABA+ (i.e. GABA + macromolecules) and glutamate. Additionally, 16 unsuppressed water scans were acquired for further water scaled metabolite quantification.

#### Metabolite quantification and quality assessment

^1^H-MRS data were pre-processed using the FID Appliance (FID-A) pipeline (www.github.com/CIC-methods/FID-A) running in MATLAB 9.2.0 (The Mathworks Inc., Natick, Massachusetts, USA). FID-A runs several steps, namely weighted receiver coil combination; removal of motion corrupted averages; frequency and phase drift correction; and spectral registration to align ON and OFF sub-spectra [50], in addition to creating the files needed for further analysis with LCModel [51] (Stephen Provencher Inc., Oakville, Canada). GABA+ (GABA plus macromolecules) and glutamate were then quantified from the difference spectrum [52] using LCModel version 6.3-1 L (http://s-provencher.com/lcmodel.shtml). The basis set used for quantification of the difference spectrum was simulated using FID-A software and high-density matrix simulations [53] with 201 × 201 × 201 spatial positions and included GABA + , Glutamate, Glutamine, N-Acetylaspartate, N-Acetylaspartilglutamate and Glutathione. Metabolite coupling constants used were based on the study by Govindaraju et al. [54], except for GABA which value was based in updated estimation from Kreis and Bolliger [55]. The unsuppressed water signal was used to obtain water-scaled metabolite values and perform eddy-current correction.

The ^1^H-MRS voxel was coregistered to the SPGR anatomical scan (see Supplementary Fig. 1) using the standalone coregistration routine from Gannet 3.0 (http://www.gabamrs.com) running in MATLAB 9.2.0 (The Mathworks Inc., Natick, Massachusetts, USA) which then runs the Statistical Parametric Mapping 12 (SPM12) (https://www.fil.ion.ucl.ac.uk/spm/software/spm12) segmentation tool to extract the proportion of gray matter (*p*GM), white matter (*p*WM) and cerebrospinal fluid (*p*CSF) within the voxel (see Supplementary Fig 3). These tissue proportion values were then used to correct the water-scaled metabolite values for partial volume effects and different amounts of ‘visible’ water in each tissue type. Each individual metabolite was corrected using the following calculation:

$${{{{{\rm{Met}}}}}}_{{{{{\rm{corr}}}}}}= {{{{{\rm{Met}}}}}}_{{{{{\rm{LCModel}}}}}}\, {{{{{\rm{x}}}}}}\, ((43300\, {{{{{\rm{x}}}}}}\, p{{{{{\rm{GM}}}}}}+35880\, {{{{{\rm{x}}}}}}\, p{{{{{\rm{WM}}}}}} +55556\, {{{{{\rm{x}}}}}}\, p{{{{{\rm{CSF}}}}}})/35880)/(1/(1-p{{{{{\rm{CSF}}}}}}))$$

where Met_corr_ is the corrected value, Met_LCModel_ is the initial LCModel output, 43300, 35880, and 55556 are the water concentrations in millimolar for GM, WM and CSF, respectively. The division by 35880 in the first fraction corrects for the LCModel initial analysis assumption of a pure WM voxel (further details in the LCModel manual, http://s-provencher.com/lcmodel.shtml). This results in the final equation:

$${{{{{\rm{Met}}}}}}_{{{{{\rm{corr}}}}}}= {{{{{\rm{Met}}}}}}_{{{{{\rm{LCModel}}}}}}\, {{{{{\rm{x}}}}}}\, (1.207\, {{{{{\rm{x}}}}}}\, p{{{{{\rm{GM}}}}}}+p{{{{{\rm{WM}}}}}} +1.548\, {{{{{\rm{x}}}}}}\, {{{{{\rm{CSF}}}}}})/(1/(1-p{{{{{\rm{CSF}}}}}}))$$

All metabolite values are reported in institutional units.

Spectra (see Supplementary Fig. 2) were visually inspected for data and fitting quality. Briefly, spectra were inspected for artifacts (subtraction artifacts, ghosts), baseline irregularities and residuals. Two participants (both with ASPD + P) were excluded from further analysis due to noisy spectra and/or poor fitting quality. Measures of spectra data quality for the final sample were: standard deviation of the Cramer-Rao lower bounds (%CRLB) between 3% and 8% for glutamate and between 3% and 5% for GABA+; signal-to-noise ratios between 20 and 31, and full width at half maximum between 0.038 ppm and 0.096 ppm.

## Results

### Demographic and clinical variables

Table 1 shows the demographic and clinical variables. As expected, the three groups differed significantly in years of education (offenders had fewer years of education than non-offenders) and PCL-R total and facet scores. There were also some differences in rates of comorbid personality disorders: offenders had a significantly higher rate of comorbid Cluster A personality disorder diagnosis compared to healthy non-offenders, and those with ASPD + P had a higher rate than those with ASPD-P. Offenders had a significantly higher rate of comorbid Cluster B personality disorder diagnosis compared to healthy non-offenders, and those offenders with ASPD + P had a higher rate than those with ASPD-P. This is in keeping with the normal range of variation in clinical profiles of ASPD +/− P and we did not adjust our analyses based on these findings. Urinary drug screening on the day of scanning revealed some significant differences in active illicit substance misuse (see Supplementary Table 1) and this was included as a covariate in supplementary ANCOVA analysis.

**Table 1:** Demographic and clinical characteristics.

	*Group*				*Group comparison (healthy non-offenders vs All ASPD)*		*Post hoc analysis (ASPD* *−* *P vs ASPD* + *P)*	
Demographic/ Clinical Characteristic^a^	Healthy non-offenders (*n* = 21)	All ASPD (*n* = 30)	ASPD − P (*n* = 16)	ASPD + P (*n* = 14)	Statistic^b^	*p* value	Statistic^c^	*p* value
Age	**35.1 (±9.8)**	**38.0 (±10.1)**	39.1 (±11)	36.7 (±9.3)	**0.993**	**0.324**	0.621	0.539
IQ	**100.3 (±9.8)**	**94.8 (±11.7)**	97.7 (±11.9)	91.3 (±10.9)	**3.017**	**0.089**	1.494	0.147
Age at first violent conviction	**n/a**	**17.6 (±7.5)**	17.7 (±8.3)	17.5 (±6.9)	**n/a**	**n/a**	0.090	0.929
Number of violent convictions	**n/a**	**6.1 (±6.8)**	5.6 (±7.9)	6.7 (±5.6)	**n/a**	**n/a**	−0.413	0.683
Years education	**14.6 (±3.3)**	**10.5 (±2.4)**	11.1 (±2.6)	9.8 (±1.9)	**25.08**	<0.001**	1.581	0.125
PCL-R Facet 1 (Interpersonal)	**0.61 (±0.92)**	**2.93 (±1.87)**	1.87 (±1.36)	4.14 (±1.65)	**26.93**	0.001**	−4.061	<0.001**
PCL-R Facet 2 (Affective)	**0.61 (±0.92)**	**4.00 (±2.01)**	2.93 (±1.52)	5.21 (±1.84)	**51.27**	<0.001**	−3.649	0.001*
PCL-R Facet 3 (Lifestyle)	**1.14 (±1.27)**	**6.50 (±1.77)**	5.56 (±1.67)	7.57 (±1.22)	**140.01**	<0.001**	−3.786	0.001*
PCL-R Facet 4 (Antisocial)	**0.42 (±0.67)**	**6.86 (±2.28)**	5.62 (±2.21)	8.28 (±1.38)	**156.21**	<0.001**	−3.994	<0.001**
PCL-R Total	**3.14 (±3.07)**	**22.44 (±6.27)**	17.51 (±3.63)	28.07 (±2.91)	**169.54**	<0.001**	−8.825	<0.001**
*Comorbid Personality Disorder Diagnosis*								
Cluster A	**0 (0%)**	**4 (13.3%)**	0 (0%)	4 (28.5%)	**–**	**0.134**	**–**	0.0365*
Cluster B	**0 (0%)**	**9 (30%)**	1 (6.25%)	8 (57.1%)	**–**	0.007*	**–**	0.0043*
Cluster C	**0 (0%)**	**2 (6.6%)**	1 (6.25%)	1 (7.1%)	**–**	0.039*	**–**	1.0
Lifetime Substance Misuse Disorder	**2 (9.5%)**	**8 (26.6%)**	5 (31.2%)	3 (21.4%)	**–**	**0.167**	**–**	0.6887

^a^Continuous data reported as mean (± standard deviation), categorical variables as raw values with (%).

^b^F_(1,49)_ for continuous variables; Fisher’s exact test for categorical variables (as all crosstab tables had at least one cell with less than 5).

^c^t statistic for continuous variables; Fisher’s exact test for categorical variables (as all crosstab tables had at least one cell with less than 5).

*statistically significant at *p* < 0.05 level; **statistically significant at *p* < 0.001 level.

ANCOVA, covarying for illicit drug use, did not lead to any changes in direction or significance of effect (striatal glutamate : GABA ratio group difference (η^2^ = 0.223, F_1,48_ = 6.876, *p* = 0.002)).

### Glutamate: GABA ratio

**Fig. 1:**
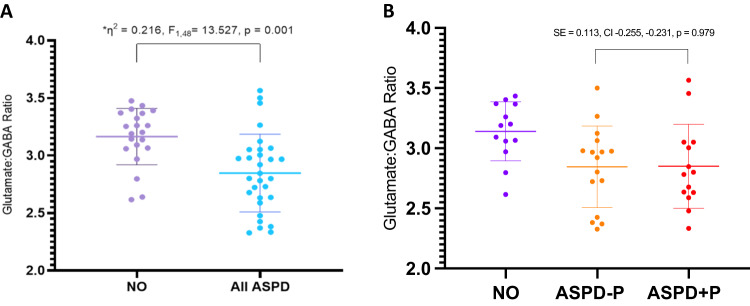
Glutamate : GABA ratios between groups. **A** Glutamate : GABA ratio within groups healthy non-offenders vs All ASPD. Individual subjects’ data plotted as dots. Means are indicated by horizontal bars. Error bars represent standard deviations. **B** Glutamate : GABA ratio within groups ASPD-P vs ASPD + P. Individual subjects’ data plotted as dots. Means are indicated by horizontal bars. Error bars represent standard deviations.

Independent sample t-test for healthy non-offenders vs All ASPD revealed a significant effect of striatal glutamate : GABA ratio between groups (SE 0.081, CI .153–0.48, *p* = < 0.001), with a lower mean ratio in All ASPD (2.847) compared to healthy non-offenders (3.164). Post-hoc comparison of ASPD-P and ASPD + P revealed no significant within ASPD group difference for striatal glutamate : GABA ratio (SE 0.113, CI −0.255–0.231, *p* = 0.979). Boxplots for the individual subject data for glutamate : GABA ratio are presented in Fig. 1. ANCOVA, covarying for illicit drug use, did not lead to any changes in direction or significance of effect (striatal glutamate : GABA ratio group difference (η^2^ = 0.223, *F*_1,48_ = 6.876, *p* = 0.002)).

### Correlation analyses

To explore the relationship between PCL-R score and Glutamate : GABA ratio in a dimensional way, we conducted a correlation analysis between PCL-R score and Glutamate : GABA ratio in both the non-offender and violent offender groups. This did not reveal any significant correlation within either the healthy non-offender group (Spearman’s rho −0.117 (−0.532 to 0.343; *p* = 0.612)) or the ASPD group (Spearman’s rho −0.076 (−0.434 to 0.302; *p* = 0.688)).

## Discussion

In a sample of violent male offenders with ASPD with and without psychopathy (ASPD +/− P), we investigated excitatory/inhibitory (E/I) striatal regulation, as measured by striatal glutamate: GABA ratio. We demonstrated impaired striatal glutamate : GABA ratio in ASPD +/− P, compared to healthy non-offenders. This finding suggests that abnormal striatal E/I balance is a shared characteristic of offenders with ASPD, and a potential cross-cutting mechanism for those with and without psychopathy. This represents a novel and important step forward towards developing a model of the neurochemical underpinnings of neurocognitive dysfunction in ASPD +/− P.

Striatal dysfunction has previously been associated with decision-making abnormalities in ASPD +/− P [37–39, 56]. Our finding of relatively increased striatal inhibitory tone in ASPD +/− P therefore provides a novel insight into potential mechanisms. For instance, inhibitory GABA-ergic spiny projection neurons likely control output from the striatum to cortical connections by their relative excitatory state [57, 58]. Increased inhibitory tone could critically impair this process. Such neuronal abnormalities would in turn have consequences at the higher cognitive level, particularly in processes related to reinforcement-based decision making. For example, should E/I regulation of ventral striatum become dysfunctional, this may lead to impaired neural reward prediction error signaling [59] and aberrant salience attribution [60]. In keeping with this, both antisocial youth [36] and adults [38] demonstrate decreased ventral striatal response during reward outcome processing, compared to controls. E/I dysregulation of the dorsal striatum could similarly interfere with the integration of information processing involved in goal-directed action and the selection of actions on the basis of their currently expected reward value [61, 62]. Supporting this, previous work in antisocial youths has demonstrated aberrant responsiveness to positive and negative prediction errors within the dorsal striatum during feedback [34], and reduced dorsal striatal response to early stimulus-reinforcement exposure [35]. Hence, impairments in both ventral and dorsal striatal function, driven by impaired E/I regulation (increased inhibitory tone), may help to explain reinforcement-based decision making impairments in ASPD +/− P.

A related consideration is whether striatal glutamate : GABA abnormalities in ASPD +/− P are primary, or if they are secondary to other factors at synaptic, circuit, or neurochemical levels. At the synaptic level, E/I balance is influenced by a number of factors, including excitatory/inhibitory synapse development, synaptic transmission, homeostatic synaptic plasticity, and intrinsic neuronal excitability. At the circuit level, E/I balance is influenced by the interplay between GABAergic interneurons and target pyramidal neurons [63], and may be related to abnormal thinning of neocortical minicolumns [64]. At a neurochemical level, glutamate and GABA’s release in vitro in the striatum are modulated by other neurochemical systems, most notably dopaminergic and cholinergic. The principal neurons in both ventral and dorsal striatum are medium-sized spiny GABA projection neurons (medium spiny neurons) that receive convergent synaptic inputs from glutamatergic and dopaminergic afferents [65]. Animal models demonstrate that activation of dopamine (D2) receptors in striatal GABAergic terminals inhibits GABA release onto cholinergic interneurons by selective blockade of N-type calcium channels [66], while dopamine modulates the excitatory glutamate corticostriatal transmission to GABA neurons [67]. Given this evidence, it is likely that synaptic and circuit factors, as well as dopaminergic and cholinergic modulation, influence the glutamate : GABA abnormalities we have demonstrated in ASPD. However, whether these interactions regulate neuromodulator levels in vivo, particularly during decision making, remains largely unknown.

Nonetheless, our findings may have implications for therapeutics in ASPD. If E/I imbalance in the striatum could be corrected, this may compensate for related neuronal dysfunction, and have beneficial effects on downstream behavioral outcomes, including aggression and sub-optimal decision-making under uncertainty. One option is to target the activity of glutamate decarboxylase (GAD), whose two isoforms convert glutamate to GABA, playing a key role in maintaining their homeostasis. Aberrant activity of GAD has been implicated in aggressive [68, 69] and social [70] behaviors [71] in animal models, and in autism [72, 73], schizophrenia [74, 75], addictions [76–78], and ADHD [79, 80] in humans. A separate promising approach may be drugs such as N-acetylcysteine and Ceftriaxone, which have been shown to normalize glutamatergic function in cocaine [81] and opiate [82] users. They do so by restoring brain production of the cystine-glutamate exchanger (xCT), which is rendered scarce by chronic drug use and leads to diminished supply of extracellular glutamate. This mechanism may be especially relevant in subjects with ASPD, who have high rates of substance dependence and abuse [83]. Similarly, psychostimulant medication may have value in ASPD. Preclinical work has demonstrated that methamphetamine may increase striatal glutamate [84] and glutamate vesicular protein concentration in dorsolateral striatum [85]. In healthy humans, both methamphetamine and d-amphetamine significantly increased glutamate (though in this case, in the anterior cingulate cortex [86]). Such an effect, should it be replicated in the striatum in ASPD, would help to normalize striatal glutamate : GABA ratio, and may have implications in attenuating related decision-making abnormalities in the condition. Future pharmacoimaging studies trialing the effect of these drugs on E/I balance may be beneficial. Selecting which specific ASPD subjects should be included in such pharmacoimaging studies is also an important consideration. A priori selection of subjects with established reduction in glutamate: GABA ratio would be the most sensible approach for interventional studies, but may be limited by its cost implications. Alternative methods are discussed in Box 1.

This work represented a methodological step forward in ^1^H-MRS studies in ASPD, in that use of 3T MRI scanner with MEGA-PRESS allowed for a direct measure of GABA (‘GABA+’), and hence a metric of E/I balance in the form of glutamate : GABA ratio, which has not been reported in this group before. Of the two previous ^1^H-MRS studies in ASPD +/− P subjects, one [87] was limited by the use of 1.5 T MRI, and reported neither glutamate or GABA levels, while the other [88] used a conventional unedited spectroscopy sequence to report only glutamate and its precursor glutamine (Glx) levels. Other key strengths of the study include clinical diagnoses and PCL-R ratings made by an experienced clinician, the use of official criminal records to classify participants, measurements of illicit substance misuse before the scan, and the use of a non-offender control group. This was a representative sample of violent offenders, with high rates of serious violence, lower than normal IQ and educational attainment, and representative histories of substance misuse.

This study has several limitations. Firstly, in common with many cross-sectional neuroimaging studies in psychiatric populations, the relatively small sample size may have resulted in smaller but important subgroup differences between the ASPD-P and ASPD + P subjects going undetected. Future studies will benefit from larger samples to determine the utility of this metric in antisocial men. Secondly, there are concerns deriving from the clinical phenotype in the disorder. Thus, significant levels of illicit drug use were observed in the antisocial men, and the drugs such as cocaine that were detected have been previously found to impact on both GABA and glutamatergic systems [89–92]. However, active substance misuse was controlled for in our analyses, and the difference between the violent and non-offending populations remained significant. Equally, our study was not designed to explore the link between Glutamate : GABA dysregulation and the neuropsychological and clinical impairments observed in the disorders. Correlation analysis did not demonstrate a significant relationship between Glutamate : GABA ratio and overall PCL-R score within the violent offender group. Both categorical and dimensional approaches thus suggest that the disruption to striatal function secondary to Glutamate/GABA dysregulation may contribute to neuropsychological impairments, such as in reinforcement learning, which are observed in both groups of violent offenders. However, such impairments were beyond the scope of our study to examine. Future studies will benefit from incorporation of appropriate neuropsychological probes to test this functional model, and to explore the potential functional impact of the observed dysregulation on subsequent recidivism [93, 94].

Finally, there are a number of technical MRS issues to consider. Although MEGA-PRESS has been demonstrated to be an acceptable means of estimating brain glutamate, the technique was not specifically designed for this purpose, and the best means of estimating the excitatory component of the glutamatergic pool continues to be debated [52, 95, 96]. A further limitation of the ^1^H-MRS procedure employed is the relatively large voxel size: striatal metabolite measures may be confounded by levels from surrounding tissue, and no distinction can be made between functional subdivisions of the striatum. Smaller voxel size however is likely to compromise the signal : noise ratio of the data [97, 98]. Furthermore, inclusion of voxels in multiple regions of interest would allow for insights into inter-relatedness of neurochemical deficits across functionally connected brain regions, for instance striatum and ventromedial prefrontal cortex. This in turn would be supplemented by combined MRS-fMRI, an emerging technique which has already demonstrated a link between Glutamatergic activity and BOLD-fMRI functional activity [99]. Finally, scanning time limitations meant that we were only able to examine a unilateral voxel of interest in the left striatum. These limitations may be addressed in future studies by using the increased spectral resolution at ultrahigh field strengths of 7 T and above, with non-edited spectroscopic techniques to address some of the identified shortcomings including signal loss and sensitivity to transmitter inhomogeneities [100].

In conclusion, we have demonstrated for the first time abnormal striatal E/I regulation in violent offenders with ASPD +/− P. This cross-cutting deficit may be a key contributor to a wider range of striatum-mediated decision-making abnormalities seen in both conditions at a behavioral level. Future studies will benefit from directly examining the relationship between Glutamate : GABA ratios in extended decision making networks and decision-making metrics derived from neuropsychological tasks, larger sample sizes, and increased precision from improved ^1^H-MRS technology. Considering the interplay of multiple neurochemical systems will be important in informing therapeutic developments in the field.

Box 1 Stratification and subject selection- the role potential of electrophysiology While stratification of violent men with ASPD into ASPD+P and ASPD-P groups is an important step forward in developing our understanding of the condition, this does have some limitations. Most notably, even with ASPD+P and ASPD-P, there will exist heterogeneity, as evidenced by the relatively wide range of values found within groups in this study. A more granular means of selecting out which specific ASPD +/− P subjects should be included in pharmacoimaging studies, and subsequent clinical trials, would therefore be beneficial. Based on the findings in this study, ASPD +/− P subjects with a striatal Glutamate : GABA ratio below a specified threshold may be selected for pharmacological intervention addressing E/I imbalance. Due to the cost and of fMRI or MRS however, using theis method to select out subjects for other studies is not pragmatic. Hence, *non*-invasive proxy measures of emotional hyporesponsivity or disrupted E/I regulation, which are linked to brain functionality, would be beneficial in several ways.

In particular, electroencephalography (EEG) may be useful. In research into ASPD +/− P, EEG has been used frequently, and several reviews have been conducted [101–103]. Together, these findings suggest significant between-group and within-group heterogeneity in ASPD +/− P on EEG parameters. EEG measures have previously been used to demonstrate atypical E/I balance in autism [104], and to link GABA dysregulation and cognitive deficits in schizophrenia [105]. Indeed, specific EEG abnormalities have been linked to specific impairments- for example activity in the gamma band in EEG recordings has been linked to impairment of dendritic GABAergic inhibition [106]. Hence, EEG has potential to be used as a proxy marker of E/I deficits in subjects with ASPD +/− P. An important first step would be to link specific EEG measures, such as P3 amplitude, to specific ^1^H-MRS abnormalities, such as Glutamate:GABA ratio in the striatum, or elsewhere.

Another potentially useful technique is Electroretinography (ERG), which measures electrophysiological activity in the retina- directly connected to the CNS via the optic nerve- and which is emerging as a useful tool for indirect investigation of brain function in psychiatric disorders [107]. For example, ERG measures have been found to be abnormal in MDD [108] and ADHD [109] (although not in Autism [110]), and evidence suggests they may be used as a marker of response to pharmacological treatment [111]. ERG measures of ganglion cell activity may be especially important, as these cells are essentially extensions of CNS axons and are modulated by both GABA and Glutamate input [112–114], [107]. Again, an important first step would be preliminary studies to link specific ERG abnormalities to specific ^1^H-MRS abnormalities, thus allowing use of ERG as a proxy measure of E/I imbalance in future work.

## Supplementary information

Supplementary Materials

## Data Availability

All data is available from the authors on reasonable request.
