# Supplementary material for: Impaired striatal glutamate/GABA regulation in violent offenders with antisocial personality disorder and psychopathy
Source: Mol Psychiatry. 2024 Feb 7;29(6):1824–32. doi: 10.1038/s41380-024-02437-4 (PMC11371654; doi:10.1038/s41380-024-02437-4)
Supplement: Supplementary file 1 — Supplementary Materials [file 41380_2024_2437_MOESM1_ESM.docx]

**S**

**A**


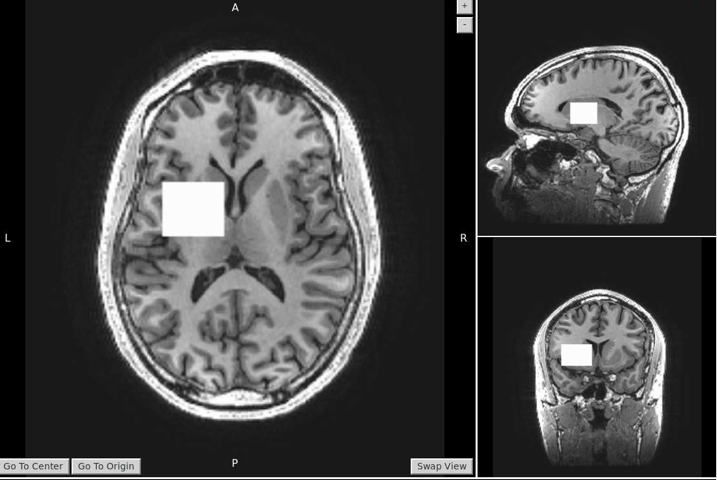

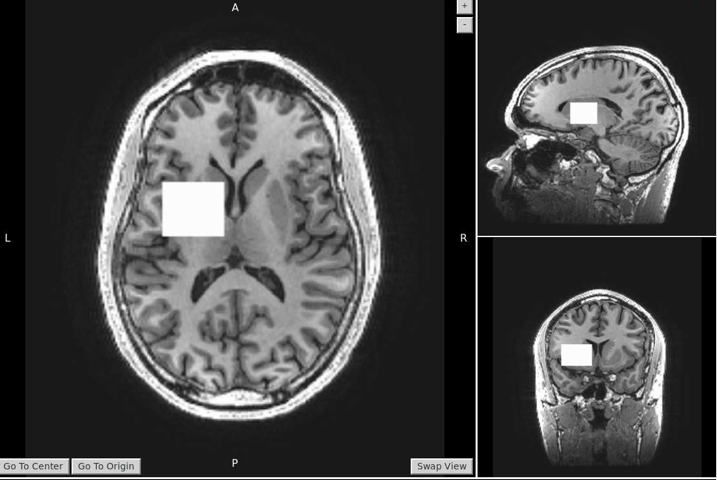


**P**

**A**

**S**

**R**

**L**

**R**

**L**

**I**

**P**

**Supplementary Figure 1. Anatomical placement of the ^1^H-MRS voxel. A, anterior; P, posterior; L, left; R, right; S, superior, I, inferior**

**
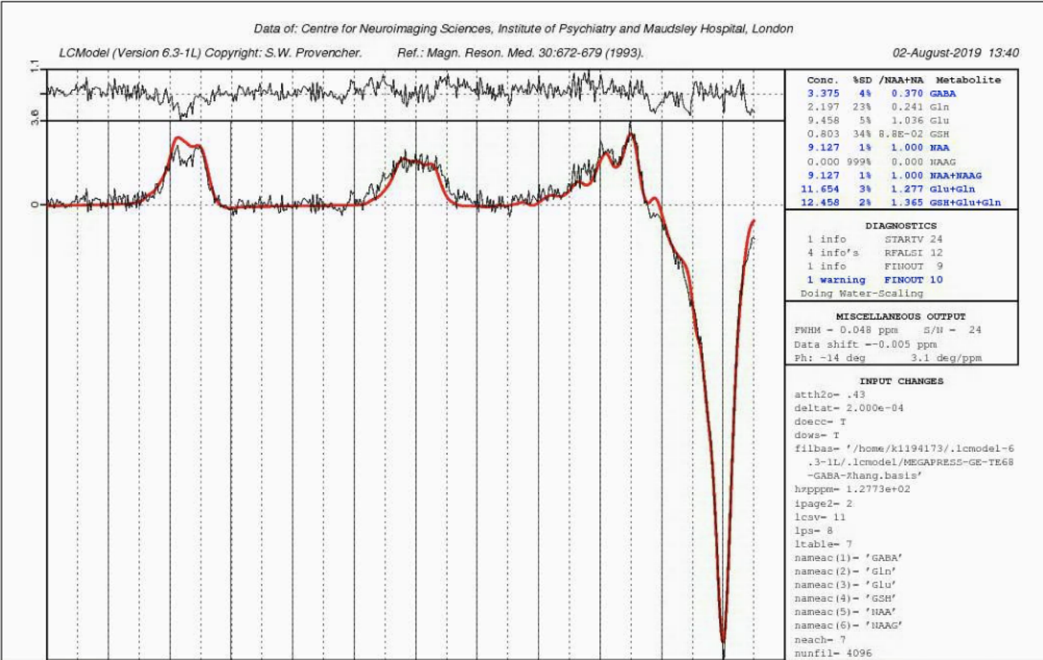
**

**Supplementary Figure 2. Example of magnetic resonance spectroscopy spectra in the ^1^H-MRS voxel**.

X axis represents the peak amplitude, and the y-axis represents the chemical shift in parts per million (p.p.m.). Abbreviations: γ-aminobutyric acid + macromolecules, GABA+; glutamate + glutamine, Glx; N-acetyl-aspartate, NAA.

**
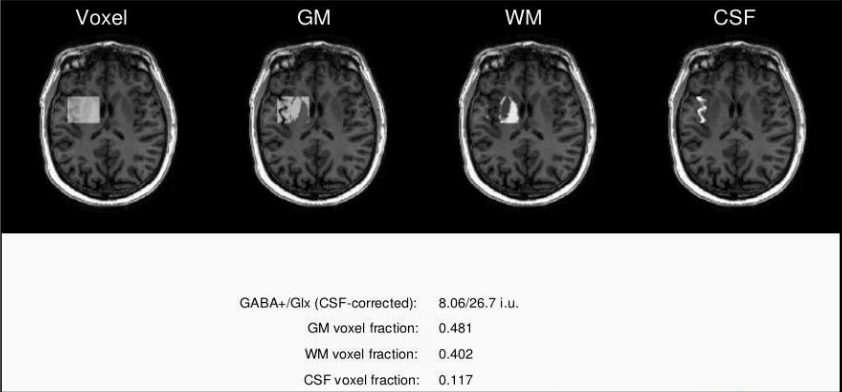
**

**Supplementary Figure 3. Example of output from segmentation step. Abbreviations: GM, grey matter; WM, white matter; CSF, cerebrospinal fluid; i.u, institutional units.**

|  | ***Group*** | | | | ***Group Comparisons**** | |
| --- | --- | --- | --- | --- | --- | --- |
| **Demographic/**  **Clinical Characteristic** | **NO**  **(n =21)** | **All ASPD**  **(n= 30)** | ASPD-P  (n= 15) | ASPD+P  (n= 19) | **NO vs All ASPD** | ASPD-P vs ASPD+P |
| **Any drug** | **4 (19%)** | **16 (53.3%)** | 8 (50%) | 8 (57.1%) | **0.019** | 0.73 |
| **Cannabis** | **4 (19%)** | **11 (36.6%)** | 5 (31.2%) | 6 (42.8%) | **0.2214** | 0.706 |
| **Cocaine** | **2 (9.5%)** | **9 (30%)** | 4 (25%) | 5 (35.7%) | **0.0977** | 0.694 |
| **Opioid** | **0 (0%)** | **3 (10%)** | 1 (6.2%) | 2 (14.2%) | **0.2588** | 0.586 |
| **Benzodiazepine** | **0 (0%)** | **3 (10%)** | 1 (6.2%) | 2 (14.2%) | **0.2588** | 0.586 |
| **Amphetamine** | **1 (4.7%)** | **0 (0%)** | 0 (0%) | 0 (0%) | **0.4118** | n/a |
| **Other hallucinogenic** | **1 (4.1%)** | **0 (0%)** | 0 (0%) | 0 (0%) | **0.4118** | n/a |

**Supplementary Table 1. Positive illicit drug tests**. *p value for Fisher’s exact test provided in each case.

1 Simpson R, Devenyi GA, Jezzard P, Hennessy TJ, Near J. Advanced processing and simulation of MRS data using the FID appliance (FID‐A)—an open source, MATLAB‐based toolkit. Magnetic resonance in medicine. 2017;77(1):23-33.

2 Provencher SW. Automatic quantitation of localized in vivo 1H spectra with LCModel. NMR in Biomedicine. 2001;14(4):260-64.

3 van Veenendaal TM, Backes WH, van Bussel FC, Edden RA, Puts NA, Aldenkamp AP, et al. Glutamate quantification by PRESS or MEGA-PRESS: Validation, repeatability, and concordance. Magnetic resonance imaging. 2018;48:107-14.

4 Zhang Y, An L, Shen J. Fast computation of full density matrix of multispin systems for spatially localized in vivo magnetic resonance spectroscopy. Medical physics. 2017;44(8):4169-78.

5 Govindaraju V, Young K, Maudsley AA. Proton NMR chemical shifts and coupling constants for brain metabolites. NMR in Biomedicine: An International Journal Devoted to the Development and Application of Magnetic Resonance In Vivo. 2000;13(3):129-53.

6 Kreis R, Bolliger CS. The need for updates of spin system parameters, illustrated for the case of γ‐aminobutyric acid. NMR in Biomedicine. 2012;25(12):1401-03.
